# Comparison of glycosylated fibronectin versus soluble fms-like tyrosine kinase/placental growth factor ratio testing for the assessment of pre-eclampsia: protocol for a multicentre diagnostic test accuracy study

**DOI:** 10.1136/bmjopen-2024-093586

**Published:** 2025-02-02

**Authors:** Nouran Elbarbary, Chao Wang, Ramesh Ganapathy, Marcus Green, Sarah Fisher, Basky Thilaganathan, Amarnath Bhide

**Affiliations:** 1 Fetal Medicine Unit, St George’s University Hospitals NHS Foundation Trust, London, UK; 2 City St. George’s, University of London, London, UK; 3 Care and Education, Kingston University, London, UK; 4 Obstetrics and Gynaecology Department, Epsom and St Helier’s University Hospitals NHS Trust, London, UK; 5 Action on Preeclampsia Charity, Evesham, UK; 6 Patient and Public Representative Advisor, London, UK

**Keywords:** Pregnancy, OBSTETRICS, Fetal medicine, Maternal medicine

## Abstract

**Introduction:**

Pre-eclampsia is a condition associated with significant maternal and neonatal morbidity and mortality. The prediction of pre-eclampsia in high-risk populations using angiogenic markers, such as serum placental growth factor (PlGF) assessment, has been shown to improve maternal outcomes and is recommended by the National Institute for Health and Care Excellence (NICE). However, such tests are not yet available at the point of care (POC). Glycosylated fibronectin (GlyFn) level for the prediction of pre-eclampsia development is available as a POC test (Lumella) and has the potential to aid rapid clinical decision making. This study aimed to test the hypothesis that the sensitivity of the GlyFn test is not inferior to that of the current gold standard of soluble fms-like tyrosine kinase (sFlt)/PlGF-based laboratory testing for pre-eclampsia.

**Methods and analysis:**

This is a multicentre prospective study. Women at risk for pre-eclampsia based on predefined clinical and/or obstetric risk factors will be invited to participate in the study. The recruitment target is 400 participants. Consenting participants will have paired samples for sFlt/PlGF together with POC GlyFn testing. Two follow-up visits are planned at 2 and 4 weeks after the initial recruitment where repeat testing with both tests will be performed. The clinical team will be blinded to the results of the GlyFn test but not that of the sFlt/PlGF test. Clinical care will be based on established protocols incorporating maternal/fetal evaluation and the results of sFlt/PlGF levels. Maternal and neonatal outcome data will be collected to compare the sensitivity and specificity of the tests, with the primary outcome being delivery for pre-eclampsia within 4 weeks.

**Ethics and dissemination:**

Ethical approval has been obtained from the Health Research Authority and Health and Care Research Wales Ethics Committee. The results of this study will be published in peer-reviewed journals and presented at scientific conferences.

**Trial registration number:**

ISRCTN13430018

**STRENGTHS AND LIMITATIONS OF THIS STUDY**

- This diagnostic test accuracy study used a paired design for the head-to-head comparison with the current gold standard test.
- The results of the Lumella test will be unknown to the clinical team and will not influence decision making.
- The clinical team will have the results of placental growth factor-based testing if available, which may introduce intervention bias.

## Introduction

 Pre-eclampsia complicates 3%–6% of pregnancies, with a 1.5-fold to a 2.0-fold higher incidence in first pregnancies and pregnancies with multiple gestations.^1^,^3^ Pre-eclampsia also has a significant impact on overall healthcare costs. A recent analysis reported that the short-term costs of pre-eclampsia in the USA in 2012 were over $2 billion.^4^ Globally, pre-eclampsia is associated with 10%–15% of all maternal deaths during pregnancy and childbirth, making it the second-leading cause of maternal mortality, resulting in an estimated 76 000 maternal deaths annually.^5 6^ Adverse maternal outcomes can be mitigated by early diagnosis, early delivery and appropriate intrapartum management. Early delivery unfortunately exposes the neonate to short-term and long-term adverse outcomes associated with prematurity. Clinical judgement and provider expertise are required to balance maternal versus neonatal risks. An effective, cost-efficient diagnostic tool would be valuable to aid decision-making. Previous studies have suggested that mortality rates could be considerably reduced if clinicians were made aware of the high likelihood of pre-eclampsia development.^7 8^

Pre-eclampsia was redefined by the American College of Obstetricians and Gynaecologists in 2013 and re-affirmed in 2019.^9 10^ Specifically, the ‘traditional’ diagnostic criteria of new-onset hypertension (>140/90 mm Hg and proteinuria >300 mg/24 hours after 20 weeks of gestation) were revised, and proteinuria is no longer required if other maternal organ dysfunction (ie, renal insufficiency, liver involvement, and neurological and haematological complications) is present. Other international studies have also added uteroplacental dysfunction or intrauterine growth restriction to the diagnostic criteria for pre-eclampsia.^11 12^ Eclampsia and the syndrome of haemolysis, elevated liver enzymes and low platelets (HELLP) can also occur in the absence of hypertension or proteinuria.^13 14^ These ‘non-traditional’ constellations of symptoms contribute to the difficulty in obtaining an accurate diagnosis of pre-eclampsia based solely on clinical criteria. The latest recommendations from the International Society for the Study of Hypertension in Pregnancy advise the use of ‘markers of angiogenic imbalance’ when evaluating women at risk for pre-eclampsia.^11^ Preterm pre-eclampsia carries a higher risk than term pre-eclampsia. It is associated with higher rates of progression to severe pre-eclampsia with an increased risk of maternal as well as fetal/neonatal morbidity and morbidity.^15 16^

An important supplement to diagnoses based on observable clinical presentation is the determination of the levels of specific biomarkers that can be measured in body fluids such as blood or urine. Several circulating factors are associated with pre-eclampsia, including soluble endoglin, placental growth factor (PlGF), soluble fms-like tyrosine kinase-1 (sFlt-1), vascular endothelial growth factor, pregnancy-associated plasma protein A-2, vasopressin, copeptin and glycosylated fibronectin (GlyFn).^17^,^21^ Although many of the above markers have shown changes in pregnancies affected by pre-eclampsia prior to onset and after the detectable onset of the condition, only some have been validated for routine clinical use to assess pre-eclampsia. A high serum level of maternal GlyFn is proposed as a highly sensitive and specific biomarker for pre-eclampsia, making it a useful adjunct diagnostic test in the evaluation of new-onset hypertension in pregnancy.^1722^,^25^ The availability as a point of care (POC) test makes it suitable for use, particularly for triage and in high-resource and low-resource settings. The Lumella GlyFn test uses a maternal finger-stick blood sample to determine the levels of a specific glycosylated form of fibronectin as a biomarker for pre-eclampsia. It has been proposed as an alternative to the sFlt-1/PlGF ratio for the assessment of the risk of developing pre-eclampsia within 2 and 4 weeks of testing.^26^ The majority of evidence for the utility of the glycosylated fibronectin test is based on retrospective studies using stored samples.

This clinical study will prospectively evaluate the quantitative POC measurement of GlyFn in maternal blood. The test will be used as an aid in the risk assessment of women with clinical findings suggestive of pre-eclampsia, in conjunction with other clinical and laboratory information. This study is designed to bridge the current gap in evidence around the effectiveness of POC GlyFn testing in the diagnosis and clinical triage for pre-eclampsia and compare performance against the current standard of care. The availability of such tests can have an impact on reducing maternal and neonatal risks associated with severe pre-eclampsia.

## Methods

### Recruitment and sampling

This prospective multicentre observational study will be conducted at St George’s Epsom and St Helier University Hospital Trusts in the UK. Recruitment started in January 2024 and is planned to last over 18 months.

The study uses predefined eligibility criteria to identify the population at risk of pre-eclampsia (table 1). Participants will undergo paired testing for the Lumella test and PlGF-based testing (Roche sFlt/PlGF test) at recruitment and in 2 and 4 weeks by the research staff. The clinical team will be blinded to POC test results. Participant information sheet and consent form are provided in onlinesupplemental files 1  2.

**Table 1:** Eligibility criteria for inclusion

Clinical risk factors (Requires on one risk factor)	
	Systolic blood pressure ≥130 mm Hg on one or more occasions
	Diastolic blood pressure ≥85 mm Hg on one or more occasions
	Elevated urinary protein
	Urine protein dipstick test 1+ or more
	Urinary protein/creatinine ratio ≥0.30 mg/mg
	Urinary protein ≥300 mg per day in timed collection
	New-onset low platelet count ≤100 000 x 109 /L
	New-onset elevated serum creatinine ≥1.0mg/dL
	New-onset transaminase elevation above the limits of normal for local laboratory
	New-onset headache unresponsive to medication and not accounted for by alternative diagnoses
	New-onset visual symptoms
	Fetal growth restriction with estimated fetal weight ≤10th percentile
Historical/obstetrical risk factors	
	History of pre-eclampsia
	Multifetal presentation
	Pre-existing hypertension
	Pre-gestational diabetes mellitus
	Pre-existing renal disease

In the UK, current NICE recommendations advise performing PlGF-based testing in cases presenting with suspected pre-eclampsia to aid in diagnosis and outcome prediction. However, test results should not be used to make decisions for delivery in isolation, and full clinical and laboratory assessment should be considered.^27^

### Inclusion and exclusion criteria

Potential participants are those with a gestational age between 24^+0^ and 36^+6^ if they are aged 18 or over with plans to deliver at the study site. The exclusion criteria are as follows: diagnosis of pre-eclampsia at the time of enrolment, planned delivery prior to 37^+0^ weeks for other clinical reasons or pregnancies with known/suspected major fetal structural or chromosomal abnormality.

### Sample size calculation

A total of 400 participants will be recruited across the study sites. Sample size of the cohort was estimated using a formula described by Alonzo *et al*^28^ for demonstrating the non-inferiority of the POC GlyFn test (Lumella) compared with PIGF and sFlt/PIGF for pre-eclampsia prediction, with sensitivity as the primary endpoint at a non-inferiority margin of 5%. The prevalence of pre-eclampsia in this high-risk population was estimated to be 23% according to data from the INSPIRE study.^29^

### Study activities and procedures

Written informed consent for participation in the study will be obtained before performing any study-specific procedures. Paired samples (fingerstick blood sample and venous blood sample in non-anticoagulated tube) will be collected at the time of enrolment and follow-up visits, where venous blood samples will be tested at the Southwest London Pathology Laboratory situated at Kingston NHS Hospital. The samples will be used for the sFlt/PlGF test with the Roche assay as is routine for participating hospitals, and an aliquot of the sample will be stored at −18 °C for future testing for serum PlGF using the POC plasma PlGF (Quidel) test retrospectively.

### Primary and secondary outcomes

The *primary outcome* is delivery for confirmed pre-eclampsia within 4 weeks of testing.

The *secondary outcomes* include diagnosis of pre-eclampsia within 7 and 28 days of sampling, severe gestational hypertension, severe preterm pre-eclampsia, severe maternal complications (cerebrovascular accidents, pulmonary oedema, renal failure or HELLP) and preterm birth.

A composite perinatal mortality/neonatal morbidity score will be assigned based on information captured including survival status at 28 days or discharge (whichever occurs first) and clinical complications including admission to the neonatal intensive care unit, respiratory distress syndrome, bronchopulmonary dysplasia, intraventricular haemorrhage, retinopathy of prematurity, necrotising enterocolitis requiring surgery and days of assisted ventilation.

All outcome data will be collected from hospital records of participants and their newborns.

## Data analysis

### Primary objective analysis

The primary analysis will be based on the cross-tabulation of the binary outcome variable, delivery for pre-eclampsia within 4 weeks of the sflt/PlGF ratio testing result versus the GlyFn (Lumella) test result. As stated, the GlyFn test results will be dichotomised into low risk/high risk based on thresholds derived from our study cohort. Sensitivity and specificity will be computed from this cross-tabulation, along with 95% CIs, which will be computed simultaneously via methods outlined by Pepe.^30^ In addition, a concordance index (C index) will be computed to measure the overall discrimination performance.^31^

To assess whether the difference between the POC GlyFn and sFLT/PlGF-based tests is statistically significant, a fixed-effects logistic regression model will be constructed given the multilevel data structure. A fixed-effects model has the advantage of taking into account any individual-level risk factors including un-observed risk factors. OR, its associated p value and 95% CI will be reported. We will also report the diagnostic performance of the POC plasma PlGF (Quidel) test for the same primary outcome.

### Secondary/subgroup analyses

Secondary analysis will estimate the positive and negative predictive values (PPV and NPV, respectively) for the detection of pre-eclampsia within 4 weeks of sampling for the POC GlyFn test, sFLT/PlGF ratio and POC plasma PlGF (Quidel) test. The PPV and NPV, along with the 95% confidence rectangle (ie, CIs for each metric estimated jointly), will be computed under various pre-eclampsia prevalence assumptions, including the prevalence observed in the current study, as well as lower/higher estimates reported in the literature. The optimum cut-off value of the Lumella test will be determined for testing the non-inferiority hypothesis for the sensitivity to the primary outcome (delivery for pre-eclampsia within 4 weeks of the first test).

Furthermore, secondary analyses will be conducted by substituting (a) pre-eclampsia within 2 weeks of sampling, (b) development of pre-eclampsia with severe features (yes/no), and (c) preterm delivery (yes/no) into the cross-tabulation instead of pre-eclampsia (yes/no).

In addition, the time to development of PE and time to delivery for subjects with low-risk and high-risk results (based on the derived test cut-off) will be assessed using Kaplan-Meier curves and Cox proportional hazards models. All analyses will be re-performed based only on certain prespecified subgroups, such as women with chronic hypertension at baseline, gestational age (<34 or ≥34 weeks), interventions and presence of gestational diabetes.

### Data management

All study documents and data will be maintained in the site’s study files.

### Protocol deviations and adverse events

All deviations from the protocol must be documented in a subject’s source documents and reported to the sponsor. The source documentation should include the reason for the deviation, attempts to correct the deviation and a plan to prevent future occurrence of the event. Adverse events must be reported to the sponsor after a discussion with the site principal investigator (PI.

### Study monitoring

At study initiation, all study-related documents will be reviewed by the sponsor representative, chief investigator and site PI. During the course of the study, AG Health will be available to the PI and study personnel to discuss adverse events, study conduct and any relevant issues pertaining to the study. During the study, a site monitor will be employed by the sponsor Advanced Global Health Limited (AGH Health).

### Trial steering committee

This includes the chief investigator, PIs for each site, trial manager, trial physician, research midwife and patient and public involvement (PPI) representatives. The committee will meet monthly to oversee trial conduct.

### Patient and public involvement

The design, study management, study monitoring and review of literature concerning participant involvement have all been aspects of public involvement in the study. The project has two PPI representatives as co-applicants for securing grant funding, ‘Katie’s team’ (a women’s health PPI advisory group) and action on pre-eclampsia (APEC, a charity registered in the UK).

### Ethics and dissemination

The Health Research Authority and Health and Care Research Wales approval has been granted for the study in October 2023. The study was given ethical approval by the London–Harrow Research Ethics Committee (IRAS ID 329734) on 13 October 2023. The study is registered at a publicly available registry (ISRCTN13430018). The results of this study will be published in peer-reviewed journals and presented at national and international conferences.

### Trial registration data

Details provided in table 2.

**Table 2:** Trial registration data

Data category	Information
Primary registry and trial identification number	ISRCTN13430018
Date of registration in primary registry	10/11/2023
Sponsor	Advanced Global Health LimitedAlex FisherAlex.fisher@agh.co.uk
Contact for public and scientific queries	Professor Amarnath Bhide.abhide@sgul.ac.uk
Scientific title	Comparison of glycosylated fibronectin test (Lumella) with the sFLT/PLGF ratio test for the assessment of pre-eclampsia
Country of recruitment	England, United Kingdom
Health condition(s) or problem(s) studied	Pre-eclampsia
Key inclusion and exclusion criteria	Age of 18 years or older Singleton or twin pregnancy Gestational age between 24^+0^ and 36^+6^ weeks Able to provide informed written consent Planned delivery at the study site or where maternal and newborn records will be available to the investigator for review Exclusion criteria: Diagnostic criteria for pre-eclampsia already met at the time of enrolment Delivery is planned prior to 37^+0^ weeks of gestation for reasons other than pre-eclampsia
Study type	Diagnostic test accuracy study
Date of first enrolment	24/01/2024
Target sample size	400
Recruitment status	Recruiting
Primary outcome	Delivery for confirmed pre-eclampsia within 4 weeks of testing, taken from participants' medical records. The sensitivity for this outcome is compared between Lumella and sFLT/PLGF as non-inferiority.
Key secondary outcomes	Pre-eclampsia diagnosed within 7 and 28 days of sampling, taken from participants’ medical records. Pre-eclampsia diagnosed in the later stage up to postpartum, taken from participants’ medical records. Results will be expressed in terms of the sensitivity and specificity of the test.

## supplementary material

10.1136/bmjopen-2024-093586 online supplemental file 1

10.1136/bmjopen-2024-093586 online supplemental file 2
