# Supplementary material for: Comparison of glycosylated fibronectin versus soluble fms-like tyrosine kinase/placental growth factor ratio testing for the assessment of pre-eclampsia: protocol for a multicentre diagnostic test accuracy study
Source: BMJ Open. 2025 Feb 2;15(1):e093586. doi: 10.1136/bmjopen-2024-093586 (PMC11792274; doi:10.1136/bmjopen-2024-093586)
Supplement: online supplemental file 1 [file bmjopen-15-1-s001.doc]

**Rec Ref:** 23/PR/0960. **IRAS ID:** 329734

Lumella test for Pre-eclampsia Study[[1]](#endnote-2)

**Participant Information Sheet (PIS)**

**Would you like to be part of a study looking at a new test for Pre-eclampsia?**

*We would like to invite you to take part in a research study. Before you decide we would like you to understand why the research is being done and what it will involve for you. Please take time to read the following carefully and discuss it with others if you wish.* ***We will go through the information sheet with you and answer any questions you have.*** *This should take about 10 minutes.*

*Please ask us if anything is not clear. Take time to decide if you wish to take part.*

**What is the purpose of the study?**

Pre-eclampsia is a risky condition, which affects approximately 5-6% of pregnancies. It is caused by a problem with the placenta and can affect many different parts of the body. The first warning signs are usually raised blood pressure and protein in the urine. However, women do not always have these symptoms and telling who is at risk from pre-eclampsia can be hard. A blood test for prediction of preeclampsia is available in the NHS, but the results can take 12-24 hours to be available. Lumella is a new test to detect pre-eclampsia and it only needs a pinprick of blood, providing results within 10 minutes. We wish to examine if this new, quick test works as well as the test currently used. Unlike Lumella, the current NHS test needs a blood sample to be sent to an off-site laboratory, meaning that women wait hours and usually overnight for their results, delaying treatment.

**Why have I been invited?**

You have been invited to participate because we think there is a chance you could be developing pre-eclampsia so we have offered you a blood test for it.

**Do I have to take part?**

No. You can decide. We will tell you about the study. If you decide to take part, please keep this information sheet and sign the consent form. You are still free to withdraw at any time and without giving a reason. This will not affect the standard of care you receive.

**What will happen to me if I take part?**

If you agree to take part, you will receive the normal care and test, but in addition to the blood sample you provide for the standard laboratory pre-eclampsia test, we will also need an extra finger-prick of blood from you. Research staff (not your clinical team providing your healthcare) will perform the new, experimental Lumella test on this blood from the finger-prick. You and your clinical team will only get the results from the standard blood test from the laboratory, not the results of the new test (the same as women who aren’t taking part in the study). The clinical team will act on the results of the standard blood test from the laboratory as usual, offering you the usual, appropriate care depending on the results.

We will ask you to return to the hospital again twice, in two and four weeks (unless you have already given birth) to repeat both tests (the usual blood sample test for the laboratory, and the extra pin-prick test). If you usually have language / translation support for your standard health appointments, this will be provided to you as usual for any additional appointments due to the study. We will provide financial assistance if you need to travel to the hospital solely for the purpose of research.

The outcome of the pregnancy will be retrieved from hospital records and you do not need to do anything else. The blood sample from the standard NHS test will be sent to the laboratory and will be stored there in the fridge to carry out pre-eclampsia tests. Your pregnancy care won’t depend on, or be affected by, the results of the new, experimental test which you and care healthcare providers won’t know the results of.

**What if I choose not to take part?**

The usual, standard blood test for detection of pre-eclampsia will be carried out anyway and your care won’t be affected.

**What are the possible disadvantages and risks of taking part?**

As well as the discomfort of the additional finger-prick test, we will repeat the test two more times (unless you have already given birth). It is likely that you will be attending the hospital for clinical reasons such as blood pressure measurements. It is unlikely that you will have to attend solely for the repeat testing.

**Will there be any additional side-effects or treatment if I take part?**

Only the discomfort / minor pain from the additional, finger-prick blood test. There are no additional/experimental treatments.

**What are the possible benefits of taking part?**

There is no direct benefit to you but you could help other women in the future by helping to check if the new test is as good or better than the current test.

**What will happen to any samples I give if I take part?**

The blood sample(s) sent to the NHS laboratory will be stored in the fridge for the duration of the study (approximately two years) to carry out pre-eclampsia tests on the samples. A different blood test for pre-eclampsia that is performed at some other trusts (but not your hospital) will also be performed on the sample(s) to compare the tests. They will be destroyed after this according to standard hospital procedures. The finger-prick blood sample will be destroyed immediately after testing.

**Will my taking part in the study be confidential and what will happen to my data? How will we use information about you?**

All your data will be kept safe and secure on NHS computers, will be accessible only to research team. People who do not need to know who you are will not be able to see your name or contact details. Your data will have a code number instead.

Personal data will not be shared with the study sponsor (AG health). Some of your information will be shared with them for the purpose of the study. These will be anonymous, and your records cannot be identified. People will use this information to do the research or to check your records to make sure that the research is being done properly. Once we have finished the study, we will keep some of the data so we can check the results. We will write our reports in a way that no-one can identify individual paricipants that took part in the study.

**What are your choices about how your information is used?**

You can stop being part of the study at any time, without giving a reason.

With your permission, we would like to keep the samples collected already but would not collect any more. If you choose to stop taking part in the study, we would like to continue collecting information about your pregnancy and your baby’s health till 28 days after birth. If you do not want this to happen, tell us and we will stop.

You also have the right to completely withdraw and not allow any of your data and the samples to be used, stored samples will then be destroyed following standard hospital protocol.

We need to manage your records in specific ways for the research to be reliable. This means that we won’t be able to let you see or change the data we hold about you.

If a participant loses the ability to consent (known as loss of capacity) during the study, all identifiable data or tissue collected would be withdrawn from the study. Data or tissue which is not identifiable to the research team may be retained. There is no plan to use these data for other future research.

**Where can you find out more about how your information is used?**

You can find out more via:

- SGHFT Privacy link:

<https://www.stgeorges.nhs.uk/education-and-research/research/research-privacy-notice/>

- For general information on how the NHS uses research data please visit <https://www.hra.nhs.uk/information-about-patients/>
- Ask a member of research team. Contact us via email: abhide@sgul.ac.uk, or Tel: 020 87250080

**What if I have a question or there is a problem?**

If there is anything about the study you are not sure of, just ask a member of your care team at the hospital. Or if you prefer speak with the researchers (Dr. Amarnath Bhide, email: abhide@sgul.ac.uk, Tel: 020 87250080). If you are unhappy about any aspect of the study and wish to make a complaint you can do this through the NHS complaints procedure. Your hospital will be able you give you information about how to do this. You can also contact the independent Patient Advice and Liaison Service (PALS) at your hospital [pals@stgeorges.nhs.uk](mailto:pals@stgeorges.nhs.uk) Tel: 020 87251609.

**Harm:**

In the event that something goes wrong and you are harmed during the research due to someone’s negligence, you may have grounds for legal action for compensation against the sonsor (Advanced Global Health Ltd), although you may have to pay your legal costs. The normal NHS complaints mechanism will still be available to you.

**What will happen to the results of the research study?**

To share the results of the study, we will present the results in scientific meetings. We also intend to publish the results in scientific journals. We will also communicate the findings of the study with members of the public and public organisations, including through the charity Action on Pre-eclampsia (APEC). We will also share results of the study with you and other participants, if you choose to receive this information by selecting this option on the study consent form.

**Who is funding, carrying out and checking the research?**

The research is funded by the National Institute for Health and Care Research (NIHR). It has been reviewed and given approval by Harrow Research Ethics Committee. The sponsor has no influence over the conduct or reporting of the study. If you have concerns, please don’t hesitate to contact us (see below for details)

**Further Information and Contact Details**

Please contact the Chief investigator Dr. Amarnath Bhide ([abhide@sgul.ac.uk](mailto:abhide@sgul.ac.uk), 020 8725 0080) if you need any additional information. Additional information and support on Pre-eclampsia is available from the charity Action on Pre-eclampsia (APEC): <https://action-on-pre-eclampsia.org.uk/>

1. Full Title: Comparison of Glycosylated Fibronectin Test (Lumella®) with sFLT/PLGF ration test for Assessment of Pre-eclampsia. Chief Investigator / Researcher: Dr. Amarnath Bhide. [↑](#endnote-ref-2)
