# Supplementary material for: Comparison of glycosylated fibronectin versus soluble fms-like tyrosine kinase/placental growth factor ratio testing for the assessment of pre-eclampsia: protocol for a multicentre diagnostic test accuracy study
Source: BMJ Open. 2025 Feb 2;15(1):e093586. doi: 10.1136/bmjopen-2024-093586 (PMC11792274; doi:10.1136/bmjopen-2024-093586)
Supplement: online supplemental file 2 [file bmjopen-15-1-s002.docx]

**REC Reference Number:** 23/PR/0960. **IRAS Project ID**: 329734

**CONSENT FORM: LUMELLA TEST FOR PRE-ECLAMPSIA STUDY**

**Title:** Comparison of Glycosylated Fibronectin Test (Lumella®) with sFLT/PLGF ratio test for Assessment of Pre-eclampsia

| **Name of Researcher:** Dr. Amarnath Bhide  Fetal Medicine Unit, St. George’s Hospital, London | | Please initial each box |
| --- | --- | --- |
| 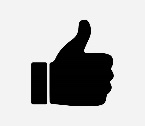I have read and understand the information about the research | |  |
| 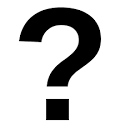 I have had the chance to think about the information, ask questions and have them answered well | |  |
|  I am happy to take part in the above study | |  |
|  I am happy for researchers to look at my medical notes | |  |
|  I understand my anonymous data will be used for the purposes of this study. No one will know my name | |  |
| I understand that all my information is stored safely   | |  |
| 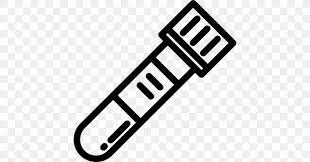I agree to my blood sample/s being used for the purposes of this study for the duration of the study, as described in the ‘Lumella Test for Pre-eclampsia Participant Information Sheet’ | |  |
|  I know I don’t have to take part. I can change my mind at any time. | |  |
|  I would like to hear the results of the study **[OPTIONAL]*.**   - *Please note this refers to final study report not individuial test results.* | |  |
| Name of participant:  Signature:  *Email address: **[OPTIONAL to receive study results]** | Date: | |
| Name of person taking consent:  Signature: | Date: | |

***When completed: Original - Investigator Site File; Copy – Participant; Copy – Medical notes***
