# Supplementary material for: Comparison of glycosylated fibronectin versus soluble fms-like tyrosine kinase/placental growth factor ratio testing for the assessment of pre-eclampsia: protocol for a multicentre diagnostic test accuracy study
Source: BMJ Open. 2025 Feb 2;15(1):e093586. doi: 10.1136/bmjopen-2024-093586 (PMC11792274; doi:10.1136/bmjopen-2024-093586)
Supplement: Supplementary file 3 [file bmjopen-2024-093586.reviewer_comments.pdf]

PEER REVIEW HISTORY

BMJ Open publishes all reviews undertaken for accepted manuscripts. Reviewers are asked to complete a checklist review form and are provided with free text boxes to elaborate on their assessment. These free text comments are reproduced below.

ARTICLE DETAILS

Title (Provisional)

COMPARISON OF GLYCOSYLATED FIBRONECTIN VERSUS SFLT/PLGF RATIO TESTING FOR ASSESSMENT OF PRE-ECLAMPSIA, PROTOCOL FOR A MULTICENTRE DIAGNOSTIC TEST ACCURACY STUDY

Authors

Elbarbary, Nouran; Wang, Chao; Ganapathy, Ramesh; Green, Marcus; Fisher, Sarah; Thilaganathan, Basky; Bhide, Amarnath

---

VERSION 1 - REVIEW

**Reviewer**	**1**
**Name**	**van der Windt, Larissa**
**Affiliation**	**Amsterdam UMC Locatie AMC, Obstetrics and Gynaecology**
**Date**	**21-Nov-2024**
**COI**	**None**

Introduction:

- I think you could elaborate more on why the current diagnostic criteria are insufficient for diagnosing pre-eclampsia. Are diagnoses missed, or is there over-diagnosis? Are they insufficient in predicting the severity of pre-eclampsia? Expanding on these points will help underline the clinical relevance of supplementing the current diagnostic methods, and therefore the relevance of your study.

- In your final paragraph, perhaps add a line about what your study will contribute to optimizing clinical practice (e.g., "Ultimately, this will result in reduced maternal morbidity and less severe pre-eclampsia.").

Methods:

- In your abstract, you state that clinicians will be blinded to the POC GlyFn test result. However, this is not mentioned in your methods section.

- Who is the sponsor?

- Why is the test repeated in 2 and 4 weeks? Will your primary outcome concern the time between the latest test moment and delivery?

- Can you elaborate on your binary outcome low-risk/high-risk? How do you make this devision?

- As healthcare systems are organized differently in each country, the methods section would benefit from a brief explanation of how pre-eclampsia care is organized at your hospital. What happens after diagnosis? What check-ups are conducted? Is PIGF already part of standard care? When is there an indication for delivery (as this is your primary outcome)? This will help other clinicians better extrapolate your results to their own practice.

- How are the perinatal outcomes diagnosed?

Minor changes:

- In abstract: change PLGF to PIGF.

- Introduction line 8 is also has?

- HRA, PI, PPI and AGH are abbreviated before providing the complete words.

**Reviewer**	**2**
**Name**	**Businge, Charles**
**Affiliation**	**University of Cape Town, Department of Medicine**
**Date**	**01-Dec-2024**
**COI**	**None**

On page 8, line 14 a term "severe preterm preeclampsia" was included.

Current opinion discourages the classification of preeclampsia as mild moderate or severe. Magee et al (2022) stressed that preeclampsia can deteriorate rapidly and without warning, which is why the ISSHP does not recommend classifying it as

non-severe or severe [Ref 11 in the manuscript].

Probably the authors intended to refer to "preterm preeclampsia with severe features".

The study commenced on 01/01/2024. In alignment with this the authors rightly stated under the section on study monitoring that "At study initiation, all study related documents were reviewed by sponsor representative, chief investigator and site principal investigator".

Later on they added that "During the study, a site monitor will either be employed by or contracted with AGH Health."

I believe the site monitor has by now been contracted or employed hence this should also be appropriately presented [see page 10, lines 6-13].

Since these are very minor revisions, I recommend to the Editor that the protocol should be published as soon as they are addressed. there is no need for me to have a second review.

VERSION 1 - AUTHOR RESPONSE

Reviewer: 1
Ms. Larissa van der Windt, Amsterdam UMC Locatie AMC

Comments to the Author:<br>Introduction:<br>- I think you could elaborate more on why the current diagnostic criteria are insufficient for diagnosing pre-eclampsia. Are diagnoses missed, or is there over-diagnosis? Are they insufficient in predicting the severity of pre-eclampsia? Expanding on these points will help underline the clinical relevance of supplementing the current diagnostic methods, and therefore the relevance of your study.
Thank you for the suggestion. This has been emphasised in the introduction. Page 4
- In your final paragraph, perhaps add a line about what your study will contribute to optimizing clinical practice (e.g., "Ultimately, this will result in reduced maternal morbidity and less severe pre-eclampsia.").
Thank you. We have added this as suggested. Page 5.
Methods:<br>- In your abstract, you state that clinicians will be blinded to the POC GlyFn test result. However, this is not mentioned in your methods section.
Thank you for your comment. This has been documented (Highlighted, Page 6).
- Who is the sponsor?
Sponsor information has been provided on P9<br>Full information given in table 2, following journal requirements (trial registration data). P15
- Why is the test repeated in 2 and 4 weeks? Will your primary outcome concern the time between the latest test moment and delivery?
Primary outcome is delivery within 4 weeks of testing. Having follow up will allow more data points to compare the head-to-head analysis of GlycFN vs standard of care test and allow the evaluation of potential change in test results over time.
- Can you elaborate on your binary outcome low-risk/high-risk? How do you make this devision?

Amended. P7. Outcome is delivery for preeclampsia within 4 weeks (yes/no). Test results obtained will be categorised at a threshold that will be defined from our cohort results to balance sensitivity and specificity and calculated from the ROC curve.
- As healthcare systems are organized differently in each country, the methods section would benefit from a brief explanation of how pre-eclampsia care is organized at your hospital. What happens after diagnosis? What check-ups are conducted? Is PIGF already part of standard care? When is there an indication for delivery (as this is your primary outcome)? This will help other clinicians better extrapolate your results to their own practice.
Thank you for the suggestion. This information has been added on P6.
How are the perinatal outcomes diagnosed?
Data on outcomes will be extracted from maternal and neonatal electronic hospital records. P7
Minor changes:<br>- In abstract: change PLGF to PIGF.<br>- Introduction line 8 is also has?<br>- HRA, PI, PPI and AGH are abbreviated before providing the complete words.
Thank you for the suggestion. The abbreviations have been explained.

Reviewer: 2
Dr. Charles Businge, University of Cape Town, Walter Sisulu University Faculty of Health Sciences

On page 8, line 14 a term "severe preterm preeclampsia" was included. Current opinion discourages the classification of preeclampsia as mild moderate or severe. Magee et al (2022) stressed that preeclampsia can deteriorate rapidly and without warning, which is why the ISSHP does not recommend classifying it as non-severe or severe [Ref 11 in the manuscript].<br>Probably the authors intended to refer to "preterm preeclampsia with severe features".
Thank you for pointing this out. We have amended as suggested.
The study commenced on 01/01/2024. In alignment with this the authors rightly stated under the section on study monitoring that "At study initiation, all study related documents were reviewed by sponsor representative, chief investigator and site principal investigator".<br>Later on they added that "During the study, a site monitor will either be employed by or contracted with AGH Health."

I believe the site monitor has by now been contracted or employed hence this should also be appropriately presented [see page 10, lines 6-13].
Thank you, has been amended. Study monitor is employed by sponsor.
Since these are very minor revisions, I recommend to the Editor that the protocol should be published as soon as they are addressed. there is no need for me to have a second review.
Thank you
